# Supplementary material for: Urinary PSA and Serum PSA for Aggressive Prostate Cancer Detection
Source: Cancers (Basel). 2023 Feb 2;15(3):960. doi: 10.3390/cancers15030960 (PMC9913326; doi:10.3390/cancers15030960)
Supplement: Supplementary file 1 [file cancers-15-00960-s001.zip › cancers-2055434-supplementary.pdf]

Supplemental Materials

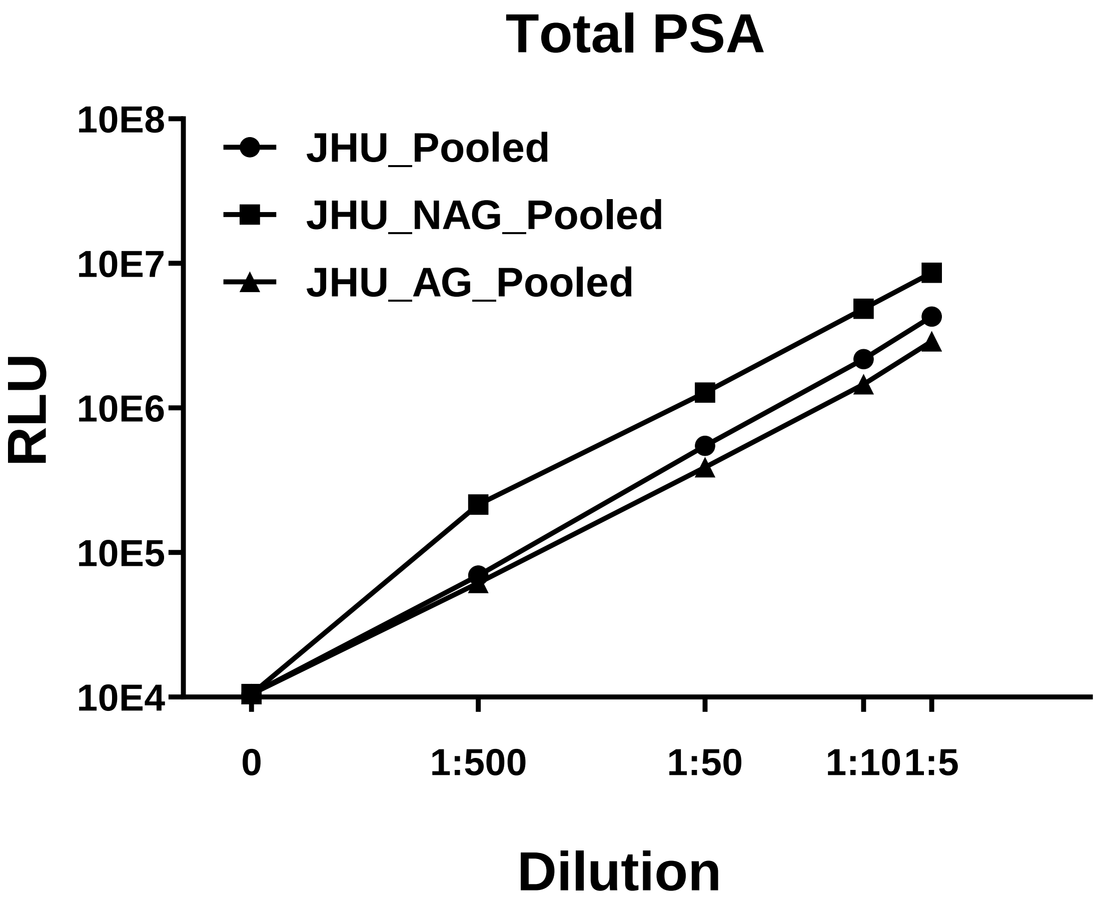

<u>Figure S1.</u> Urine PSA dilution: To determine the dilution factor to perform the urinary PSA assay; equal volumes (10ul) from the 146 urine samples were pooled (JHU_Pooled) or 76 non-aggressive samples (JHU_NAG_Pooled) or 70 aggressive samples (JHU_AG_Pooled) and then diluted 500 folds for total PSA analysis.

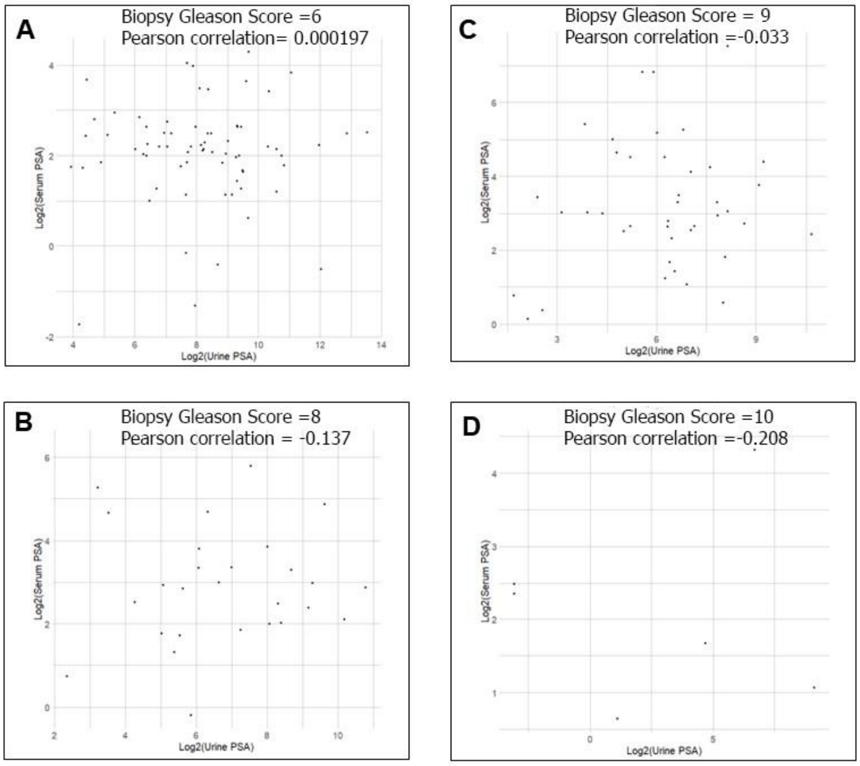

E

Correlation b/w Urine and Serum PSA

	Pearson correlation
G6	0.000197
G8	-0.137
G9	-0.033
G10	-0.208

2

Figure S2. Correlation between Serum and Urinary PSA: In aggressive prostate cancer, higher PSA values were detected in the serum samples of the patients, these patients however, had lower level of urinary PSA. A Pearson correlation analysis was performed across all the RRP Gleason scores (6, 8, 9, 10) to see any negative association between the two biological (serum vs urine) specimens within the same patients (A, B, C, D). The Pearson correlation demonstrated no significant association between the samples. Statistical significance was defined at $p \leq 0.05$.

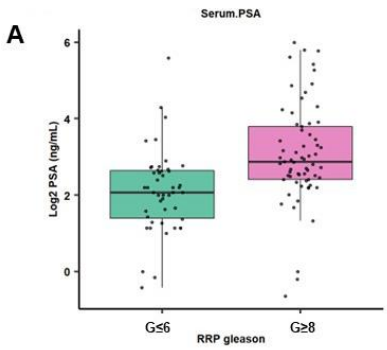

RRP Gleason	p-value
G≤6 vs G≥8	5 x 10-5

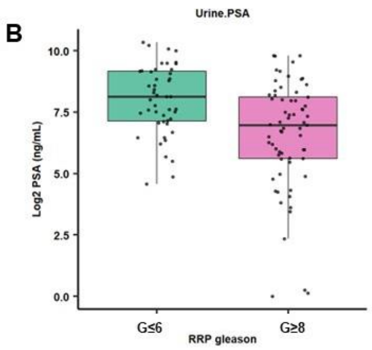

RRP Gleason	p-value
G≤6 vs G≥8	3.7 x 10-4

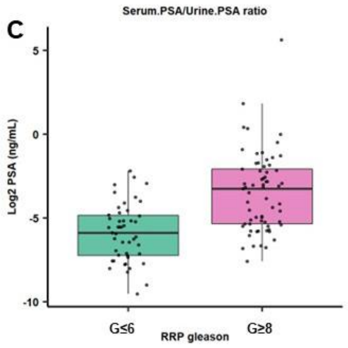

RRP Gleason	p-value
G=6 vs G=8	1.9×10^{-7}

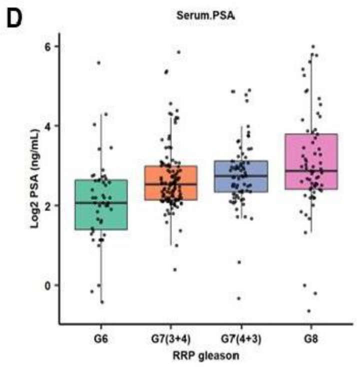

RRP Gleason	p-value
G6 vs 3+4	3.2×10^{-4}
G6 vs 4+3	2.9×10^{-5}
G6 vs G7	2.6×10^{-5}
G6 vs G8	5.0×10^{-6}
3+4 vs 4+3	0.086
3+4 vs G8	2.7×10^{-3}
4+3 vs G8	0.159

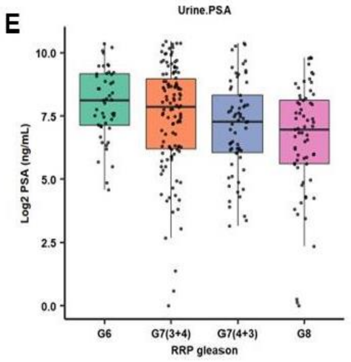

RRP Gleason	p-value
G6 vs 3+4	0.288
G6 vs 4+3	8.1×10^{-3}
G6 vs G7	0.065
G6 vs G8	3.7×10^{-4}
3+4 vs 4+3	0.094
3+4 vs G8	4.9×10^{-3}
4+3 vs G8	0.327

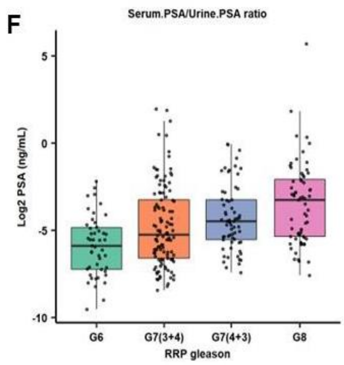

RRP Gleason	p-value
G6 vs 3+4	0.011
G6 vs 4+3	3.4×10^{-5}
G6 vs G7	4.9×10^{-4}
G6 vs G8	1.9×10^{-7}
3+4 vs 4+3	0.051
3+4 vs G8	2.9×10^{-4}
4+3 vs G8	0.070

Figure S3: Utility of serum to urine PSA ratio in predicting the pathological Gleason outcomes: The box and whisker plots showing the median PSA values and overall dispersion of individual PSA values for serum (A) or urine (B) and their respective ratio in samples with surgical Gleason GS 6 vs GS 8 samples (C). The box plots were used to stratify the serum and urine PSA (D, E) and the ratio of serum to urine PSA (F) across different pathological grades (RRP) along with their *p* -values.

Table S1. cohort 1.

Specimen.Number	Urine.PSA	Serum.PSA	RRP_Class	Biopsy_Class	Specimen.Number	Urine.PSA	Serum.PSA	RRP_Class	Biopsy_Class	Specimen.Number	Urine.PSA	Serum.PSA	RRP_Class	Biopsy_Class	Specimen.Number	Urine.PSA	Serum.PSA	RRP_Class	Biopsy_Class	Specimen.Number	Urine.PSA	Serum.PSA	RRP_Class	Biopsy_Class
1	18.24	0.3	G1	G1	35	179.4	3.4	G1	G1	69	21.24	5.4	G1	G1	103	20.76	8	G4	G4	137	25.44	31.8	G4	G4
2	247.56	0.4	G1	G1	36	1844.04	3.43	G1	G1	70	1634.52	5.4	G2	G4	104	8.76	8.1	G4	G4	138	64.56	36.1	G4	G4
3	4194	0.7	G1	G1	37	268.2	3.5	G4	G4	71	34.44	5.5	G1	G1	105	15.12	8.11	G4	G4	139	111.6	38.4	G4	G4
4	412.32	0.75	G1	G1	38	457.56	3.58	G1	G1	72	0.12	5.6	G4	G4	106	283.08	8.3	G4	G4	140	9.36	38.6	G4	G4
5	58.2	0.87	G4	G4	39	29.88	3.6	G1	G1	73	144.96	5.6	G1	G1	107	99.72	9.8	G4	G4	141	14.28	42.3	G4	G4
6	202.32	0.9	G1	G1	40	152.4	3.6	G4	G4	74	317.52	5.6	G4	G4	108	225.96	9.8	G4	G4	142	186.24	55.5	G4	G4
7	4.32	1.1	G4	G4	41	206.04	3.6	G1	G1	75	330.48	5.6	G1	G1	109	411.72	9.8	G4	G4	143	18.6	79.99	G4	G4
8	5.88	1.3	G4	G4	42	619.2	3.9	G1	G1	76	356.04	5.6	G1	G1	110	67.56	10.1	G4	G4	144	60.24	112.7	G4	G4
9	256.68	1.5	G4	G4	43	83.28	4	G1	G1	77	7551.6	5.6	G1	G1	111	127.32	10.2	G4	G4	145	47.76	113.2	G4	G4
10	815.88	1.54	G1	G1	44	268.68	4	G4	G4	78	124.2	5.66	G1	G1	112	1299.36	10.7	G1	G1	146	283.68	183.4	G4	G4
11	2.16	1.56	G4	G4	45	662.52	4	G1	G1	79	19.32	5.7	G4	G4	113	5.28	10.75	G4	G4					
12	5.16	1.66	G4	G4	46	1735.68	4	G1	G1	80	32.16	5.7	G4	G4	114	331.08	11	G1	G1					
13	3.24	1.7	G4	G4	47	336.96	4.05	G4	G4	81	11798.4	5.7	G1	G1	115	275.64	11.21	G1	G1					
14	89.04	2	G1	G1	48	77.4	4.1	G1	G1	82	131.16	5.8	G4	G4	116	101.4	11.22	G4	G4					
15	120	2.1	G4	G4	49	492.6	4.12	G1	G1	83	80.76	6.2	G4	G4	117	781.68	12.5	G1	G1					
16	551.16	2.1	G4	G4	50	212.16	4.2	G1	G1	84	82.8	6.2	G1	G1	118	21.72	12.8	G1	G1					
17	201	2.2	G1	G1	51	365.52	4.2	G1	G1	85	251.28	6.2	G1	G1	119	545.88	13.6	G4	G4					
18	491.04	2.2	G1	G1	52	292.68	4.3	G1	G1	86	646.68	6.2	G1	G1	120	67.68	13.9	G4	G4					
19	570.84	2.2	G1	G1	53	1160.64	4.3	G4	G4	87	700.92	6.2	G1	G1	121	2150.4	14.3	G1	G1					
20	1553.52	2.3	G1	G1	54	64.44	4.4	G1	G1	88	141.12	6.27	G4	G4	122	257.4	14.4	G4	G4					
21	76.32	2.35	G3	G4	55	301.32	4.4	G1	G1	89	36.925	6.3	G4	G4	123	237	15.8	G1	G1					
22	104.28	2.4	G1	G1	56	1544.04	4.4	G1	G1	90	639	6.3	G1	G1	124	207.12	16.5	G1	G1					
23	699.24	2.4	G1	G1	57	110.52	4.6	G1	G1	91	401.16	6.59	G4	G4	125	131.04	17.4	G4	G4					
24	41.64	2.5	G4	G4	58	132.24	4.6	G1	G1	92	131.64	6.7	G1	G1	126	195.72	18.9	G4	G4					
25	93.72	2.7	G4	G4	59	224.88	4.6	G1	G1	93	81.84	6.92	G4	G4	127	823.68	19.6	G1	G1					
26	637.32	2.7	G1	G1	60	1273.08	4.6	G1	G1	94	25.56	7	G1	G1	128	103.56	19.9	G4	G4					
27	727.44	3.12	G1	G1	61	283.32	4.68	G1	G1	95	49.32	7.2	G4	G4	129	595.2	21	G4	G4					
28	718.92	3.18	G1	G1	62	4015.2	4.7	G1	G1	96	70.68	7.2	G1	G1	130	22.44	21.02	G4	G4					
29	25.8	3.2	G4	G4	63	84.96	4.77	G1	G1	97	1764	7.28	G4	G4	131	37.08	22.8	G4	G4					
30	84	3.2	G4	G4	64	307.56	4.9	G1	G1	98	33.72	7.6	G4	G4	132	75.84	22.8	G4	G4					
31	19.68	3.3	G1	G1	65	87.24	5	G4	G4	99	40.56	7.7	G1	G1	133	27.72	24.95	G4	G4					
32	46.44	3.3	G3	G4	66	517.92	5	G1	G1	100	229.44	7.7	G4	G4	134	11.52	25.4	G4	G4					
33	15.24	3.36	G1	G1	67	0.12	5.1	G4	G4	101	621.48	7.9	G4	G4	135	80.28	25.78	G4	G4					
34	32.76	3.4	G4	G4	68	577.92	5.2	G3	G4	102	99.72	7.95	G3	G4	136	792.24	29.1	G4	G4					

Table S2. cohort 2.

Specimen.Number	Urine.PSA	Serum.PSA	RRP_Class	Biopsy_Class	Specimen.Number	Urine.PSA	Serum.PSA	RRP_Class	Biopsy_Class	Specimen.Number	Urine.PSA	Serum.PSA	RRP_Class	Biopsy_Class	Specimen.Number	Urine.PSA	Serum.PSA	RRP_Class	Biopsy_Class	Specimen.Number	Urine.PSA	Serum.PSA	RRP_Class	Biopsy_Class
1	1.2	0.64	G4	G4	36	137	3.6	G4	G4	70	217	4.4	G2	G2	104	48.3	5.07	G4	G3	138	598	5.8	G3	G2
2	381	0.75	G1	G1	37	72.9	3.7	G2	G3	71	449	4.4	G2	G3	105	400	5.1	G2	G2	139	19.5	5.8	G4	G4
3	26.5	0.8	G3	G3	38	712	3.76	G1	G1	72	202	4.42	G3	G1	106	432	5.1	G2	G1	140	78.7	5.9	G2	G2
4	56.9	0.87	G4	G4	39	242	3.76	G3	G3	73	64.2	4.5	G2	G2	107	211	5.1	G3	G3	141	219	5.9	G2	G2
5	197	0.9	G1	G1	40	762	3.8	G2	G2	74	1327	4.5	G2	G1	108	531	5.1	G3	G2	142	403	5.9	G2	G1
6	739	1	G1	G1	41	1	3.9	G2	G2	75	230	4.5	G3	G3	109	77.9	5.2	G2	G1	143	263	5.9	G4	G4
8	289	1.31	G2	G2	42	143	4	G1	G1	76	161	4.53	G2	G2	110	79.1	5.2	G2	G2	144	330	5.91	G4	G4
9	20	1.5	G3	G3	43	176	4	G1	G1	77	170	4.57	G4	G4	111	411	5.2	G2	G2	145	19.3	5.93	G2	G2
10	73.7	2	G1	G2	44	601	4	G1	G1	78	498	4.58	G4	G1	112	115	5.2	G3	G2	146	8.23	5.94	G2	G2
11	115	2	G2	G2	45	834	4	G2	G2	79	131	4.6	G1	G1	113	587	5.2	G3	G4	147	1052	5.94	G2	G4
12	51.2	2.2	G1	G2	46	319	4.05	G4	G4	80	149	4.6	G1	G1	114	279	5.2	G4	G4	148	242	5.95	G2	G2
13	134	2.2	G1	G1	47	195	4.1	G2	G2	81	217	4.6	G1	G1	115	23.9	5.26	G1	G2	149	45.6	6	G1	G2
14	166	2.2	G1	G1	48	394	4.1	G2	G2	82	1193	4.6	G1	G1	116	189	5.3	G2	G1	150	457	6	G1	G1
15	467	2.2	G1	G1	49	1407	4.1	G2	G2	83	52.6	4.6	G2	G3	117	19	5.3	G4	G4	151	75.9	6	G2	G1
16	570	2.2	G1	G1	50	48	4.1	G3	G3	84	215	4.6	G2	G1	118	217	5.3	G4	G4	152	614	6.1	G2	G1
17	723	2.4	G1	G1	51	568	4.14	G2	G1	85	393	4.6	G2	G2	119	122	5.4	G2	G2	153	29.6	6.1	G4	G4
18	88.3	2.44	G1	G2	52	193	4.2	G1	G1	86	72	4.6	G3	G3	120	172	5.4	G2	G3	154	538	6.2	G1	G1
19	44.4	2.5	G4	G4	53	290	4.2	G1	G1	87	130	4.6	G3	G3	121	174	5.4	G2	G1	155	605	6.2	G1	G1
20	385	2.58	G1	G2	54	577	4.2	G1	G1	88	880	4.6	G4	G4	122	366	5.5	G2	G1	156	423	6.2	G2	G2
21	587	2.6	G2	G2	55	59.6	4.2	G2	G2	89	20.7	4.7	G2	G3	123	86.6	5.5	G3	G3	157	1024	6.3	G1	G1
22	582	2.7	G1	G1	56	378	4.2	G2	G2	90	797	4.7	G2	G3	124	12.3	5.5	G4	G3	158	2.61	6.3	G2	G4
23	258	3	G1	G1	57	180	4.2	G3	G4	91	1264	4.7	G2	G1	125	1.51	5.54	G2	G1	159	383	6.3	G2	G2
24	334	3	G2	G1	58	438	4.29	G2	G1	92	257	4.7	G4	G4	126	155	5.6	G3	G3	160	90.4	6.3	G4	G4
25	184	3.1	G1	G1	59	145	4.3	G2	G2	93	796	4.76	G2	G1	127	249	5.6	G4	G4	161	102	6.4	G1	G2
26	444	3.18	G1	G1	60	264	4.3	G2	G4	94	81.8	4.77	G1	G1	128	126	5.64	G3	G3	162	11.7	6.4	G3	G3
27	162	3.2	G3	G3	61	320	4.3	G2	G3	95	1009	4.8	G2	G1	129	1082	5.7	G1	G1	163	199	6.4	G4	G4
28	115	3.2	G4	G4	62	325	4.3	G2	G2	96	191	4.8	G3	G4	130	259	5.7	G2	G2	164	18.1	6.5	G2	G2
29	58.7	3.3	G3	G3	63	974	4.3	G2	G1	97	115	4.8	G4	G4	131	146	5.7	G3	G2	165	278	6.5	G2	G2
30	65.5	3.3	G3	G3	64	84	4.3	G3	G3	98	17.8	4.9	G2	G2	132	691	5.7	G3	G3	166	413	6.5	G2	G3
31	154	3.4	G2	G2	65	90.6	4.3	G3	G3	99	608	4.9	G2	G3	133	196	5.7	G4	G4	167	988	6.5	G2	G2
32	27.3	3.4	G4	G4	66	30.6	4.36	G2	G4	100	226	4.9	G3	G2	134	574	5.7	G4	G4	168	5.1	6.51	G4	G4
33	45.3	3.5	G2	G2	67	598	4.39	G2	G2	101	13	5	G2	G2	135	576	5.77	G2	G2	169	17.5	6.56	G3	G2
34	677	3.5	G2	G1	68	21.3	4.4	G2	G3	102	283	5	G2	G1	136	54.7	5.8	G2	G1	170	53.9	6.59	G4	G4
35	29.2	3.6	G1	G1	69	111	4.4	G2	G2	103	34.7	5.06	G3	G2	137	33.5	5.8	G3	G3	171	88.3	6.6	G1	G1

Table S2. cohort 2 continue

Specimen.Number	Urine.PSA	Serum.PSA	RRP_Class	Biopsy_Class		Specimen.Number	Urine.PSA	Serum.PSA	RRP_Class	Biopsy_Class		Specimen.Number	Urine.PSA	Serum.PSA	RRP_Class	Biopsy_Class		Specimen.Number	Urine.PSA	Serum.PSA	RRP_Class	Biopsy_Class
172	25.2	6.6	G2	G1		207	441	7.9	G4	G4		241	282	11	G1	G1		275	471	23.6	G2	G1
173	153	6.6	G3	G1		208	890	7.9	G4	G4		242	73	11	G2	G1		276	74.1	24.8	G3	G3
174	138	6.7	G1	G1		209	75.5	7.95	G3	G4		243	832	11	G2	G3		277	73.4	25.8	G4	G4
175	195	6.7	G1	G1		210	45.2	8	G2	G1		244	246	11	G3	G1		278	461	27.6	G3	G3
176	468	6.7	G2	G3		211	1063	8	G2	G4		245	599	11	G4	G2		279	178	29.1	G3	G1
177	22.6	6.7	G3	G3		212	1323	8	G2	G3		246	79.9	11.1	G2	G2		280	893	29.1	G4	G4
178	632	6.7	G3	G2		213	1115	8	G3	G3		247	16.8	12	G4	G4		281	74.6	29.9	G3	G3
179	8.93	6.79	G3	G3		214	135	8.17	G4	G4		248	474	12.2	G3	G3		282	64.3	30	G4	G4
180	316	6.8	G1	G1		215	69.4	8.2	G2	G1		249	825	12.4	G3	G3		283	11	38.6	G4	G4
181	156	6.8	G2	G3		216	1194	8.2	G2	G2		250	1293	12.6	G2	G2		284	292	40.5	G2	G3
182	687	7	G2	G2		217	24.2	8.3	G3	G1		251	37	12.8	G2	G2		285	29.5	41.7	G2	G2
183	62.5	7	G4	G4		218	14.1	8.4	G2	G3		252	31.5	13	G4	G3		286	162	42.9	G4	G4
184	71.3	7.1	G3	G3		219	230	8.4	G2	G2		253	41.5	13.4	G3	G3		287	280	48	G1	G3
185	63.2	7.12	G4	G4		220	170	8.4	G3	G3		254	59.6	13.5	G2	G2		288	290	49	G4	G4
186	793	7.2	G2	G2		221	762	8.4	G3	G1		255	1229	13.5	G3	G3		289	1.1	54.9	G4	G4
187	168	7.28	G4	G4		222	251	8.4	G4	G1		256	58.7	13.9	G4	G4		290	180	55.5	G4	G4
188	179	7.3	G2	G2		223	394	8.44	G2	G3		257	15.1	14.3	G3	G3		291	457	64	G4	G4
189	192	7.3	G2	G2		224	184	8.5	G2	G3		258	295	14.4	G4	G4						
190	751	7.35	G4	G4		225	111	8.6	G2	G4		259	126	14.7	G4	G4						
191	238	7.37	G2	G3		226	83.6	8.7	G3	G3		260	94.2	15	G4	G4						
192	1158	7.4	G2	G2		227	366	8.7	G4	G1		261	1277	15.9	G3	G4						
193	190	7.4	G3	G3		228	6.43	8.8	G2	G2		262	183	16.5	G1	G1						
194	274	7.4	G3	G3		229	467	9	G4	G3		263	714	17	G2	G1						
195	344	7.43	G1	G2		230	89.7	9.26	G2	G3		264	702	17.9	G2	G2						
196	70.9	7.6	G3	G3		231	198	9.3	G3	G3		265	128	17.9	G4	G4						
197	233	7.6	G3	G3		232	66.7	9.42	G3	G3		266	65.2	18.4	G2	G2						
198	19.9	7.6	G4	G4		233	107	9.5	G4	G4		267	512	18.5	G2	G1						
199	567	7.7	G3	G3		234	394	9.7	G4	G3		268	14.1	18.8	G4	G4						
200	1330	7.7	G3	G3		235	76.4	9.89	G4	G4		269	717	19.6	G1	G1						
201	49.4	7.7	G4	G4		236	10.4	9.9	G3	G3		270	55.1	19.6	G2	G2						
203	320	7.8	G3	G2		237	1307	10.7	G1	G1		271	149	19.9	G2	G3						
204	403	7.8	G3	G3		238	379	10.7	G3	G3		272	153	20	G4	G4						
205	79.2	7.86	G2	G2		239	48.7	10.7	G4	G4		273	77.3	21	G2	G2						
206	40.8	7.9	G2	G4		240	29.5	10.8	G3	G3		274	104	23.2	G4	G4						

Table S3. Description of prostate cancer tissues used in the immune-histochemistry staining for PSA.

Metastases	Gleason score of primary tumor				PSA	
	7	*8*	>8	N/A	Pos	Neg
Lung (10)	2	5		3	4/6	2/6
Bone (10)	2	4	1	3	3/7	4/7
Dis LN (7)	1	4	1	1	3/5	2/5
Pleural effusion (6)	1	3	1	1	4/5	1/5
Liver (6)	2	2		2	1/4	3/4
Bladder (5)	2	2		2	0/2	2/2
Loc LN (4)	2	1		1	N/A	N/A
Adrenal (1)			1		1/1	0/1
Others (12)	3	4	1	4	3/3	0/3
Total*	15	25	5	17		

Table S4 The clinical information of these cases and the Gleason scores of primary tumors.

Description of prostate cancer tissues used in immune-histochemistry staining of PSA.

Clinical characteristics	Cases (%)
Age	47-84 yrs, median 72.5
Caucasian	10 (16.1)
Africa American	52 (83.9)
Smoking Status	
Former/Current	48 (77.4)
Never	4 (6.5)
Unknown	10 (16.1)
Treatment prior to metastasis	
Prostatectomy only	17 (27.4)
Surgery-chemo	10 (16.1)
Chemotherapy only	16 (25.8)
Radiation only	1 (1.6)
Radiation-chem	8 (12.9)
Unknown	10 (16.1)

Supplemental Table S3 & S4: Description of prostate cancer tissues used in the immune-histochemistry staining for PSA and the clinical information of these cases and the Gleason scores of primary tumors.
